# Effects of inhibiting mTOR with rapamycin on behavior, development, neuromuscular physiology and cardiac function in larval *Drosophila*

**DOI:** 10.1242/bio.046508

**Published:** 2019-11-21

**Authors:** Samuel Potter, Jacob Sifers, Emily Yocom, Sandra L. E. Blümich, Rachel Potter, Jeremy Nadolski, Douglas A. Harrison, Robin L. Cooper

**Affiliations:** 1 Deptartment of Biology and Center for Muscle Biology, University of Kentucky, Lexington, KY, 40506, USA; 2 Alice Lloyd College, 100 Purpose Road, Pippa Passes, KY, 41844, USA; 3 Kentucky Wesleyan College, Owensboro, KY, 42301, USA; 4 Veterinärmedizinische Fakultät, Universität Leipzig, Leipzig, Germany; 5 Department of Mathematical and Computational Sciences, Benedictine University, Lisle, IL, 60532 , USA

**Keywords:** Synapse, Heart, Skeletal muscle, Behavior

## Abstract

Rapamycin and other mTOR inhibitors are being heralded as possible treatments for many human ailments. It is currently being utilized clinically as an immunomodulator after transplantation procedures and as a treatment for certain forms of cancer, but it has numerous potential clinical indications. Some studies have shown profound effects on life cycle and muscle physiology, but these issues have not been addressed in an organism undergoing developmental processes. This paper fills this void by examining the effect of mTOR inhibition by rapamycin on several different qualities of larval *Drosophila*. Various dosages of the compound were fed to second instar larvae. These larvae were monitored for pupae formation to elucidate possible life cycle effects, and a delay to pupation was quantified. Behavioral deficits were documented in rapamycin-treated larvae. Electrophysiological measurements were taken to discern changes in muscle physiology and synaptic signaling (i.e. resting membrane potential, amplitude of excitatory post-synaptic potentials, synaptic facilitation). Pupation delay and effects on behavior that are likely due to synaptic alterations within the central nervous system were discovered in rapamycin-fed larvae. These results allow for several conclusions as to how mTOR inhibition by rapamycin affects a developing organism. This could eventually allow for a more informed decision when using rapamycin and other mTOR inhibitors to treat human diseases, especially in children and adolescents, to account for known side effects.

## Abstract (precis)

**Summary:** Inhibiting mTOR by rapamycin delays pupation, reduced body wall contractions and mouth-hook movements while synaptic transmission appeared normal in larval *Drosophila.*

## INTRODUCTION

Regulating growth and division is an important feature of all cells, both within unicellular and multicellular organisms. As such, cells have multiple biochemical systems that regulate the process. One such system is the mTOR (mechanistic target of rapamycin) pathway, named after its inhibitor (i.e. rapamycin; Guertin et al., 2006). First discovered on Easter Island in the 1970s, rapamycin was initially noteworthy for its antibiotic properties in inhibiting the growth of fungus (Vezina et al., 1975). Subsequent studies in other organisms, particularly yeast, found that rapamycin prevented progression through the cell cycle. This led to the discovery of the mTOR nutrient-sensing pathway and its role in cellular growth and division (Barbet et al., 1996). Since then, the mTOR pathway has been found to be highly conserved in a variety of organisms and rapamycin has consistently been effective at inhibiting mTOR (Jacinto and Hall, 2003).

The protein at the center of the mTOR pathway is TOR kinase, which functions in two complexes, TORC1 and TORC2. Of the two, TORC1 seems to be the complex that is sensitive to rapamycin inhibition (Loewith et al., 2002). As such, much more is known of its biochemical function. Studies have shown that it is active in nutrient-rich environments, which leads to phosphorylation of downstream effectors such as S6K. S6K appears to be important in promoting cellular growth and metabolism, as organisms deficient in this protein show similar growth deficits to low-caloric intake (Bjedov et al., 2010).

Rapamycin has recently been heralded as a potential avenue of treatment of psychiatric and neurologic conditions as more research has shown the importance of the mTOR1 pathway in neurologic processes. Activation of the mTORC1 signaling pathway has been shown increase glutamate receptors in hippocampal cells and play an important role in memory and learning in mice (Hsu et al., 2019). With the relatively newfound interest of studying mTOR in neuronal physiology, the potential therapeutic use of rapamycin and other mTOR inhibitors in neuropsychiatric and other diseases has also increased (Hasty, 2010; Heard et al., 2018; Hosoi et al., 1998; Stern and Berliner, 2019; Qiao et al., 2019).

Since mTORC1 plays an important role in the cell cycle, one would expect inhibition by rapamycin to affect organismal life cycle, for life cycle progression is a direct result of the processes of cellular growth and division. Work done in organisms ranging from unicellular yeast (Kaeberlein et al., 2007) to mammals (Selman et al., 2009) have found that mTORC1 inhibition arrests life cycle progression. In adult fruit flies (*Drosophila melanogaster*), mTORC1 inhibition by rapamycin was found to significantly increase life span (Bjedov et al., 2010), but the effect in the developing larval system was not addressed. No previous physiological or behavioral measures have been obtained related to alterations in synaptic function by rapamycin treatments in adults or larval *Drosophila*. Due to the documented effect of rapamycin on halting progression through the cell cycle, we hypothesized that larval *D. melanogaster* that are fed rapamycin will progress more slowly through their life cycle and have a delayed time to pupation in a dose-response pattern with higher dosages of rapamycin resulting in a longer pupation delay.

Synaptic transmission between neurons or motor neurons and skeletal muscle is an energy-demanding process and coordinated by many proteins. Driving the system with higher demand, such as with repetitive stimulation, can uncover abnormalities in the synaptic process. Using short-term facilitation (STF) by repetitive stimulation is well established from insects to mammals and is a process dependent on the handling of Ca^2+^ influx within the presynaptic terminal. Residual cytoplasmic calcium within the terminal, due to previously evoked activity, can accumulate, resulting in an increase in the amount of transmitter released (Katz and Miledi, 1968; Sherman and Atwood, 1971). The residual [Ca^2+^]_i_ is altered by Ca^2+^ binding proteins and their dissociation rates (Augustine, 2001; Kretsinger and Nockolds, 1973; Lundh, 1998; Sheng et al., 1998). Ca^2+^ pumps and the sodium/calcium exchanger (NCX) can rapidly regulate [Ca^2+^]_i_ and thus effect the rise and decay of [Ca^2+^]_i_ (see reviews: Berridge, 1997, 2005; Berridge et al., 2000; Budde et al., 2002; Desai-Shah and Cooper, 2009; Friel and Chiel, 2008; Thayer et al., 2002). All these proteins regulating ion balance and vesicle fusion process have particular turnover and synthesis rates (Brockhaus et al., 2019).

The neuromuscular junction (NMJ) of larval *Drosophila* is an ideal preparation to investigate STF within the presynaptic nerve terminal in regard to the efficacy of synaptic transmission since the excitatory junction potentials (EJPs) are graded. In addition, innervation of the one or two excitatory motor neurons to a single large target cell (i.e. a muscle fiber) provides an ideal response to examine if decreasing protein synthesis by rapamycin within defined time windows has an effect that can be correlated with behavioral changes. Surprisingly, little attention has been focused on the potential effects of rapamycin on synaptic transmission and STF in model preparations.

At the *Drosophila* larval NMJ, synaptic transmission is enhanced or depressed depending on how the [Ca^2+^]_i_ load is managed within the presynaptic terminal during STF (Wu and Cooper, 2012). Thus, we used a STF stimulation paradigm in larvae fed rapamycin for 24 h to ascertain any subtle effects on synaptic transmission in addition to the single stimulus responses of EJPs.

Studies have shown that mTOR activation is important in the development and maintenance of skeletal muscle fibers (Bodine et al., 2001). In rodents, for example, mTORC1 inhibition leads to decreased muscle protein synthesis and delayed heart development (Drummond et al., 2009). The importance of mTOR on skeletal muscle development has not, however, been examined in a developing organism. The mTORC1 pathway has been found to be important in maintaining cardiac function in certain disease states (Shende et al., 2016), the effects of mTOR inhibitors as part of medical treatment regimens can take a toll on cardiac function (Eldahan et al., 2018), and inhibiting mTORC1 complex in mice resulted in high mortality within 6 weeks (Shende et al., 2011). Biomarkers have been identified to help clinicians to be aware of such adverse effects as they arise with patients receiving rapamycin treatments (Witteles, 2016).

Heart disease and cardiac translational research is increasingly coming from the *D. melanogaster* model. The genes involved in heart development and the molecular mechanisms of cardiac function are similar between *D. melanogaster* and humans (Bodmer, 1995; Cripps and Olson, 2002; Na et al., 2013; Nishimura et al., 2011; Wessells et al., 2004). Thus, we examined the effect of acute rapamycin treatment on the cardiac function in larval *Drosophila* at various doses. As a bioindex, we used heart rate and the change in heart rate to a cardiac modulator [serotonin (5-HT)] as an additional measure since it is known that 5-HT can increase the larval heart rate. The larval heart uses a 5-HT2 receptor subtype mediated through G-protein coupled receptors and a PLC-PKC pathway, resulting in a rise in intracellular release of Ca^2+^ from stores (Majeed et al., 2014). Rapamycin is known to inhibit Ca^2+^ reuptake by SERCA in some cell types (Bultynck et al., 2000), and it is established that SERCA is important for cardiac regulation in *Drosophila* larvae, as well as in mammals (Armoundas et al., 2007; Desai-Shah et al., 2010; Heitner and Hollenberg, 2009; Trafford et al., 2009). We reasoned that in the rapamycin-treated larvae the increase in heart rate by 5-HT might be dampened.

In addition, due to the known role of mTOR in skeletal muscle maintenance, we hypothesized that larval *Drosophila* fed rapamycin will have neuromuscular abnormalities that can be quantified using behavioral assays and physiological measurements.

## RESULTS

### Life cycle quantification

**Fig. 1.**
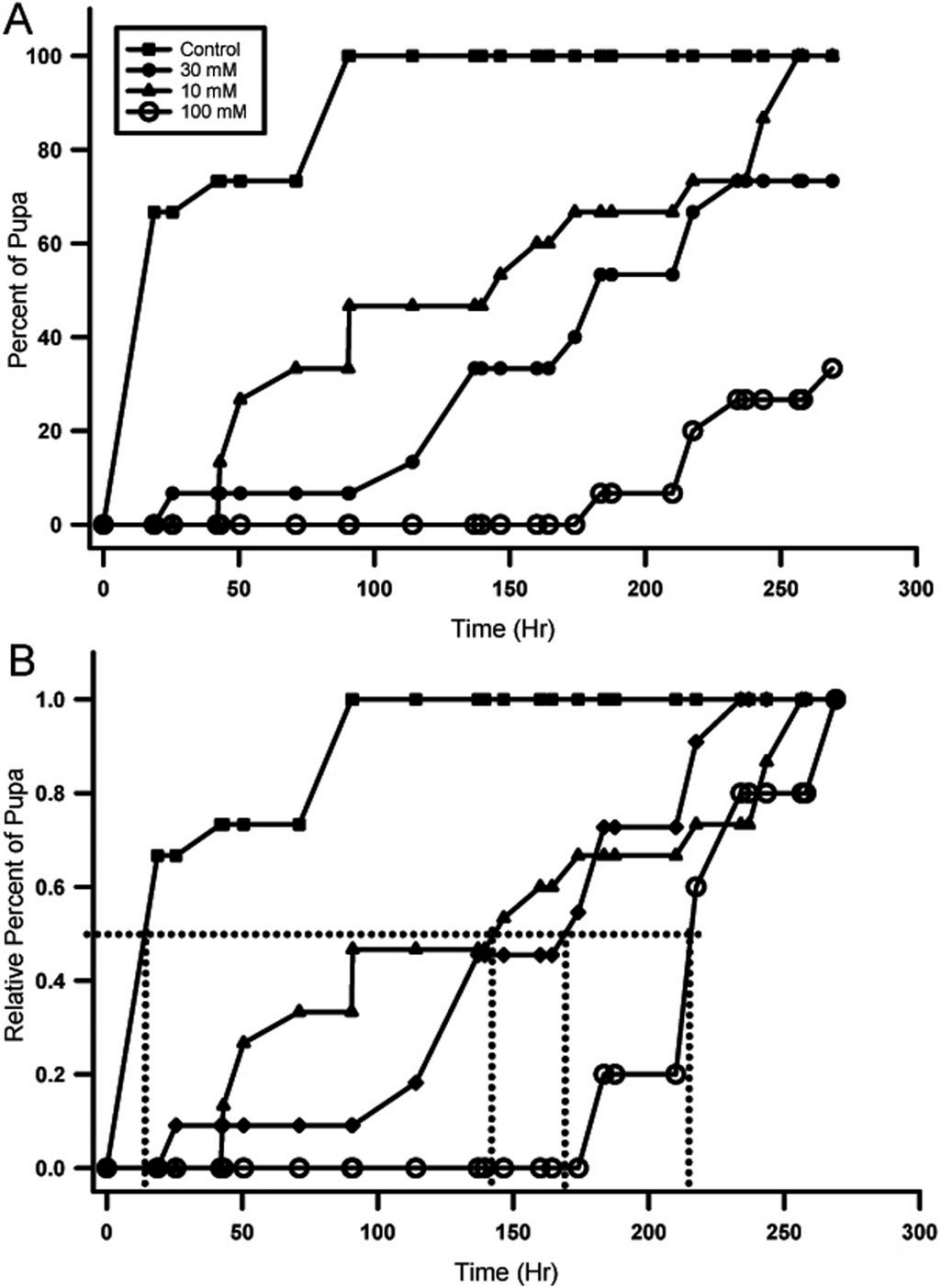
**Rate of pupation for control and rapamycin treatments.** (A) The rate of pupation from first instar to pupa. For each time period assessed, a percentage of the total number of starting larvae was determined. The higher the concentration of rapamycin in the food, the greater the lag time in becoming a pupa. Even after 250 h (∼10 days) larvae were still alive in the food but did not develop into a pupa. (B) To better visualize the shift in pupation time, a relative percentage of those that did pupate is shown. Note the time taken for half of those that did pupate. There is a markedly prolonged time to pupation for rapamycin treatments. The 10 and 30 µM treatments are similar; however, the 100 µM treatment is greatly extended in time, and all treatment groups are significantly prolonged when compared to control.

Data from the life cycle quantification aspect of the project consisted of a proportion of larvae that pupated over time. The percentage of the 15 larvae in each group that pupated over time is shown (Fig. 1A). The two higher dosages (30 µM and 100 µM) did not reach 100% pupation at the end of data collection, but that was not due to larval death. Larvae were still alive, crawling and eating in both vials at the end of data collection. A log-rank statistical analysis comparing pupation rate of each experimental group to that of control found that all three experimental pupation rates were significantly different from the control rate at an alpha level of 0.05 (*P*<0.001). A relative percentage of pupated larvae is also shown to normalize the total number (Fig. 1B).

### Behavioral assays

**Fig. 2.**
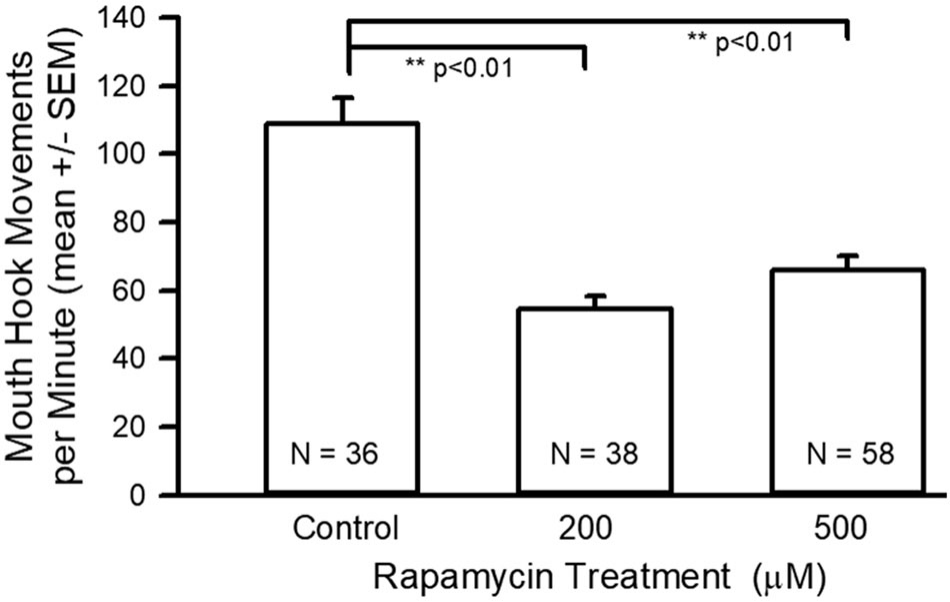
**Mouth-hook movement assay data for control and rapamycin treatments.** There was a significant reduction in the mouth-hook movements for the rapamycin-fed larvae (ANOVA, **P<0.01).

Data from the behavioral assays consisted of rates of mouth-hook movements and body-wall movements for each of the three groups. The average number of mouth-hook movements in 1 min for each of the three groups is shown. The rate was reduced for 200 and 500 µM treatments compared to controls (Fig. 2; ANOVA *P*<0.01). The 200 and 500 µM-fed groups were not different from each other.

**Fig. 3.**
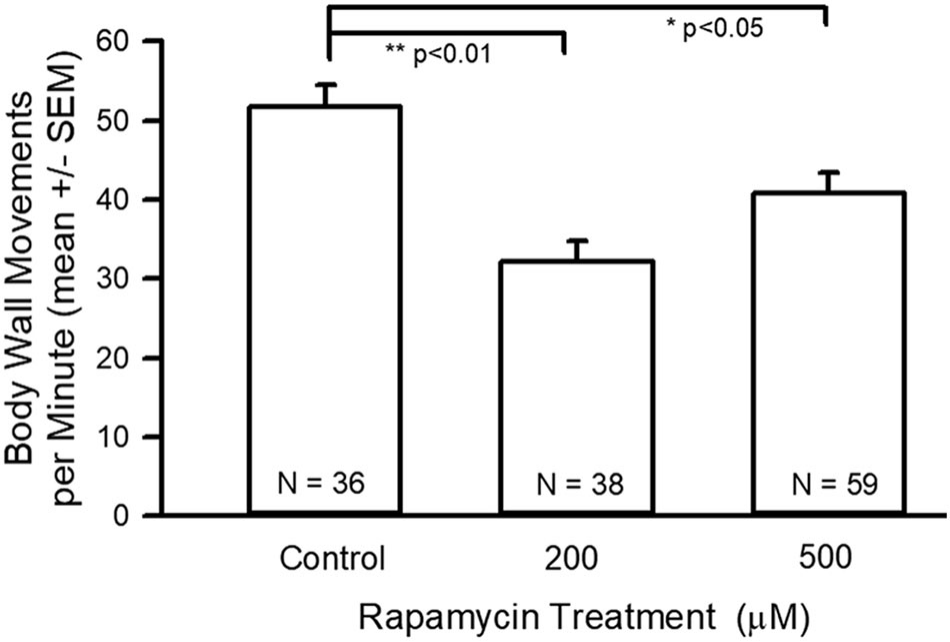
**Body-wall movement assay data for control and rapamycin treatments.** There was a significant reduction in the body-wall movements for the 200 μM (ANOVA, **P<0.01) and 500 μM (ANOVA, *P<0.05) rapamycin-fed larvae in comparison to control.

The average number of body-wall movements in 1 min was statistically significant for the 200 and 500 µM-fed larvae from the control (*P*<0.01 and *P*<0.05, respectively), but the two experimental groups were not significantly different from one another (Fig. 3).

Mechanical stimuli on the cuticle of the larvae can elicit a series of typical behaviors, which have been previously described (Kernan et al., 1994; Titlow et al., 2014; Zhong et al., 2010). A light tactile stimulus (20 mN) was given to the three regions for the HAT assay (head, abdomen and tail) with the sharp end of an insect pin. Notice in all cases there was a large increase in no responses for larvae fed the high concentration of rapamycin (500 μM, Table 1).

**Table 1.**
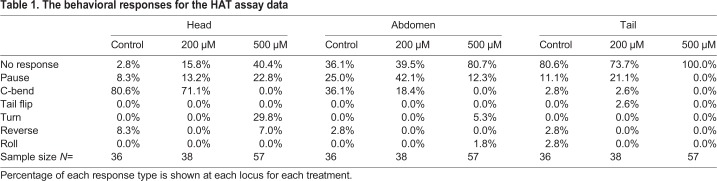
**The behavioral responses for the HAT assay data**

### Electrophysiological recordings

**Fig. 4.**
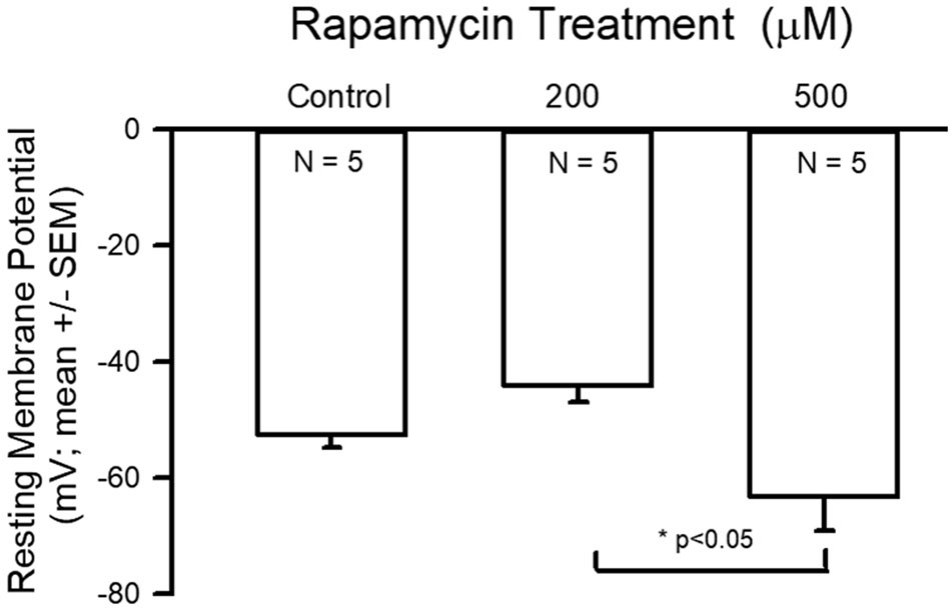
**Resting membrane potential data for control and rapamycin treatments.** The 200 µM-treatment group had a more depolarized potential than the 500 µM group (ANOVA, *P<0.05).

There was no difference in the resting membrane potential among treatments compared to control; however, the 200 µM-treatment group had a more depolarized potential than the 500 µM group (Fig. 4; ANOVA, *P*<0.05).

**Fig. 5.**
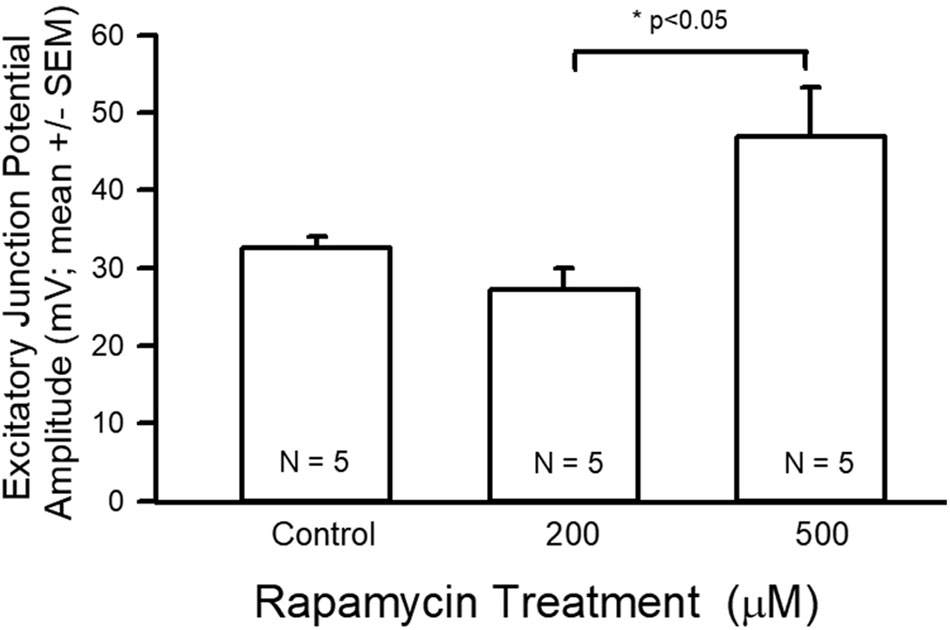
**EJP amplitude data for control and rapamycin treatments.** EJP amplitude was significantly larger for the 500 µM group as compared to the 200 µM group (ANOVA, *P<0.05).

**Fig. 6.**
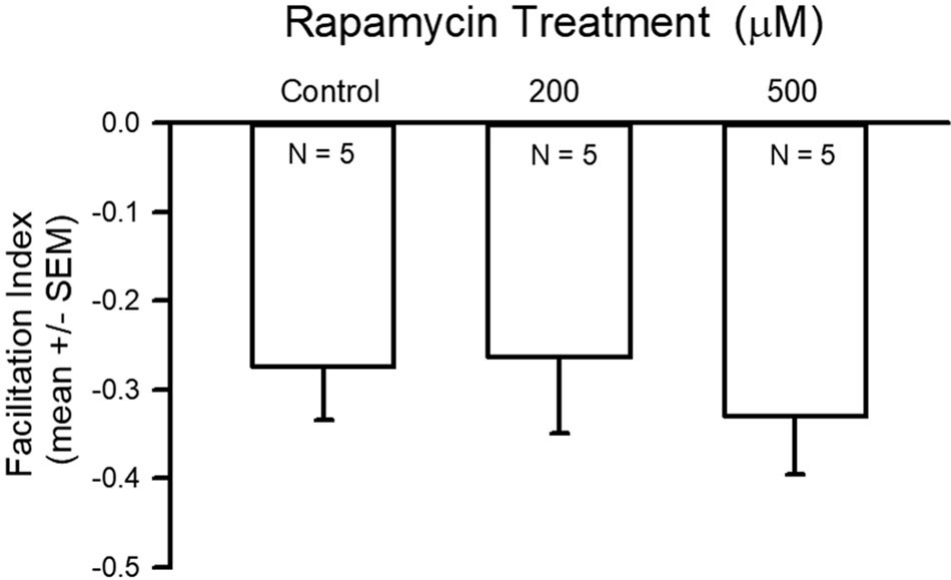
**Facilitation index of EJPs.** Facilitation index of EJPs stimulated at 40 Hz for four pulses was not different among the treatment and control groups.

The average EJP amplitude was larger for the 500 µM group as compared to the 200 µM-fed group but neither of the treatment groups were different from the control group (Fig. 5; ANOVA, *P*<0.05). The average facilitation index across all three groups was not significantly different, although more variation was seen within the 200 µM treatment than within the control or 500 µM treatments. (Fig. 6).

**Fig. 7.**
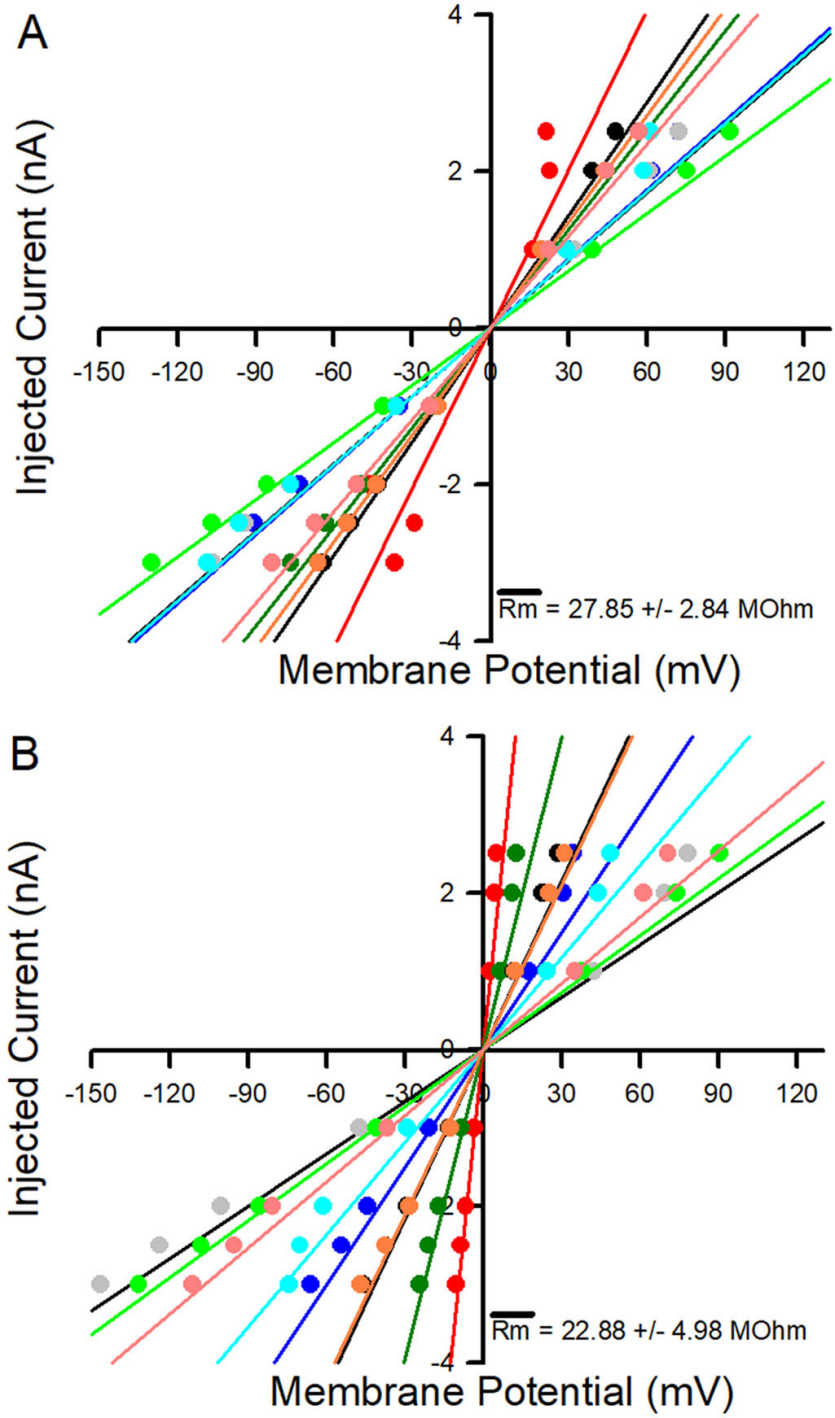
**Input resistance of muscle fibers.** Current versus potential data for control (A) and rapamycin (B) treatments. The mean membrane resistance (Rm) (+/−s.e.m.) is given for each condition. There was no significant difference in the input resistance between the control and 200 µM feed rapamycin groups.

The input resistance of the muscle fibers of larvae was examined to assess the integrity of the skeletal muscle membrane. Since 200 µM-fed rapamycin groups had the greatest impact of body-wall movements and mouth-hook movements, this treatment was examined. The current versus membrane potential is shown for each preparation in the two conditions (Fig. 7). There was no significant difference in the input resistance between the groups; however, the variation was greater for the rapamycin-treatment group.

### Heart rates

**Fig. 8.**
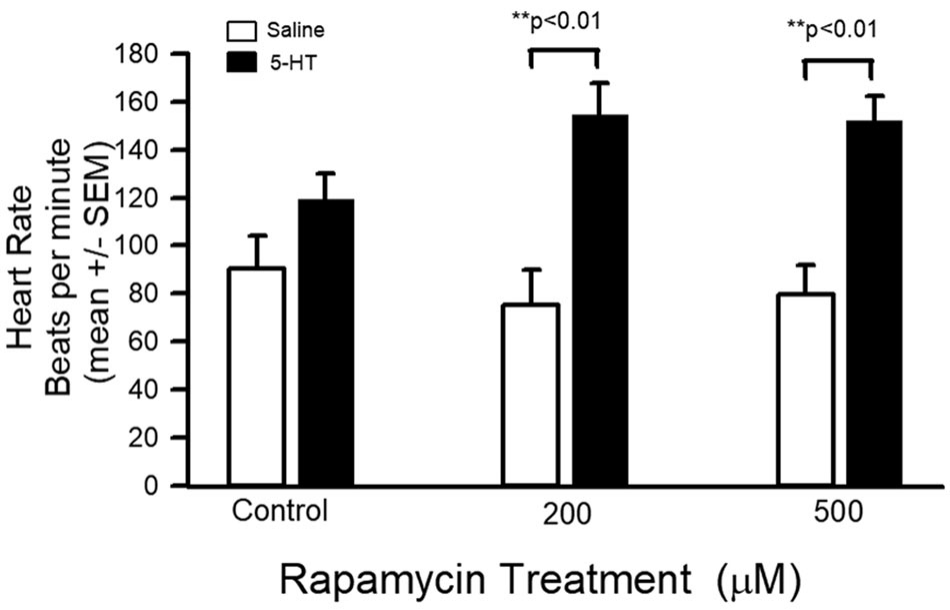
**Heart rate data for control and rapamycin groups in saline and 5-HT treatments.** Both 200 and 500 µM rapamycin-treated larvae showed substantial increases in heart rate following addition of 5-HT (100 nM; paired *t*-test **P<0.01).

The average heart rate in 1 min for each of the three experimental groups is shown. The 200 and 500 µM treatments show a large, significant increase in heart rate following addition of 5-HT (100 nM) (Fig. 8; paired *t*-test *P*<0.01).

## DISCUSSION

In this study, we showed a reduction in the rate of development for the larval *Drosophila* as noted for other animal species exposed to rapamycin. A significant delay to pupation occurred for all three rapamycin treatments when compared to the ethanol control. Additionally, there was a dose-response relationship between rapamycin and time to pupation, with higher dosages taking longer to pupate. These results are consistent with findings in adult *D. melanogaster*, which showed that rapamycin treatment led to an extension of life span (Bjedov et al., 2010). Though consistent, the findings herein are unique in that they are applicable to a developing larval system. In addition, reduced body-wall contractions, mouth-hook movements and responses to mechanical stimuli are consistent with the possibility that neural circuits are not responding in a normal manner with ingestion of rapamycin. Examining the synaptic transmission and input resistance of the skeletal muscle did not reveal any deficits that could account for the altered behaviors. The cardiac function was normal and maybe even slightly enhanced when exposed to the cardiac modulator 5-HT. These results do not suggest a systemic wide defect, but one more specific to the central circuits that coordinate the behaviors we examined.

The results from the behavioral assays were also consistent with work done in other studies that showed that mTORC1 inhibition by rapamycin led to behavioral alterations; however, these were due to muscle abnormalities in rodents (Drummond et al., 2009). Since these behavioral assays serve as proxies for muscle health and neural coordination, decreased performance indicates issues with control of skeletal muscle. In addition, the slowed larval development leads to smaller body size and shorter segmental skeletal muscles. The skeletal muscles appear to function well for receiving synaptic transmission and depolarization, but we have not tested muscle function in E–C coupling and the ability of force development, which was beyond the scope of this study. Interestingly, the mouth-hook and body-wall assays found that intermediate dosages of rapamycin had the most profound effect on behavior, with the 200 µM treatment having the largest reduction in movement in relation to control. The lack of a dose-response relationship may indicate mTOR desensitization to high dosages of rapamycin or an increase of clearance of the compound.

The results from the electrophysiological experiments have some interesting implications. Though rapamycin treatment did lead to a significant change to resting membrane potential when comparing experimental groups to the control, the pattern is not dose-dependent. In fact, the intermediate dosage of 200 µM led to an increase in resting membrane potential, and the high dosage of 500 µM led to a decrease in resting membrane potential. These findings were paralleled in the EJP amplitude, which showed that the intermediate dosage had a significantly smaller amplitude than the high dose. The EJP amplitude in control larvae, which was in the middle of the two experimental groups, was not significantly different from either group. Together with the results from the behavioral assays, these results indicate that the intermediate dosage of 200 µM of rapamycin has a greater effect on NMJ physiology than the higher dose. However, it appears that handling of intracellular Ca^2+^ with repetitive motor nerve stimulation is not substantially altered by treatment of rapamycin, since there is no significant difference in the facilitation-depression index. The EJP amplitudes are also robust with even the high concentration exposure to rapamycin.

We have found the larval heart to be very sensitive to variations in intracellular Ca^2+^ dynamics (Desai-Shah et al., 2010; Zhu et al., 2016a) and modulation by biogenic amines (Anyagaligbo et al., 2019; Dasari and Cooper, 2006; Dasari et al., 2007; Majeed et al., 2013; Malloy et al., 2016; Titlow et al., 2013) in previous studies. 5-HT in particular increases the heart rate (Majeed et al., 2014; Zhu et al., 2016b) and has direct action on the heart as the pacemaker region in early third instar heart does not have neuronal innervation (Johnstone and Cooper, 2006). Since the response in an increase in heart rate to 5-HT exposure is pronounced for the larvae fed rapamycin, it would appear that the PLC-PKC pathway in the heart is not negatively compromised. It is established that this cellular pathway is the predominate mechanism for the mechanistic action of 5-HT on the larval heart (Majeed et al., 2014). Given that there is a greater increase in heart rate with 5-HT exposure beyond the control (ethanol-exposed larvae), this might indicate that maybe there is an alteration in the heart cells to tightly regulate Ca^2+^ by the SERCA, NCX or Ca-pump when stressed by exposure to modulation, which likely rises intracellular Ca2+. Since rapamycin can inhibit Ca^2+^ reuptake by SERCA in some cell types (Bultynck et al., 2000) more detailed studies are needed to address this possibility in the *Drosophila* larval heart.

The question remains as to the physiologic mechanism by which the intermediate rapamycin dosage impacts larval behaviors and growth. Unfortunately, rapamycin is not water-soluble, so an organic solvent must be used to bring it into solution. There does not appear to be an issue with muscle membrane being leaky to ions or depolarization of resting membrane potential, since membrane resistance and synaptic response are not compromised due to the solvent or rapamycin. The preparations used as controls address the point that ethanol as a solvent is not an issue; however, ethanol may present some limitation to this study if one wanted to try higher concentrations of rapamycin. Early trials conducted in the lab found that the quantities of solvent required limited the options to ethanol, as DMSO proved to be highly lethal to the *Drosophila* larvae. The 48-h evaporative period for ethanol worked well for survival of the larvae. However, ethanol has effects on the systems being examined. For example, studies have shown that ethanol also affects muscle development and protein metabolism (Steiner and Lang, 2015). Some studies have found that ethanol also inhibits mTOR, resulting in the same net effect of treatment with rapamycin (Mazan-Mamczarz et al., 2015). As such, it is difficult to tease out muscle abnormalities due to mTOR inhibition by rapamycin from abnormalities caused by mTOR inhibition by ethanol and ethanol's effects not related to mTOR. Future studies could potentially address this matter.

For the direct effect of rapamycin on the altered behaviors, the likely target is in the central circuits within the central nervous system (CNS) of the larvae. This is not so readily addressed in behaving larvae, but one can approach this in isolated *in situ* preparations (Dasari and Cooper, 2004a,b). As for retarding larval growth and time to pupation, this may also be due to alteration in CNS function of hormonal control, which is essential for larval growth and development. For example, if ecdysone production is dampened by reduced neural endocrine regulation then larval development will be delayed (Li et al., 2001).

## CONCLUSIONS

Behavioral deficits and slowed development occur when larvae are fed rapamycin. Neuromuscular physiology appears normal; however, potential effects on cellular mechanisms of muscle contraction still need to be addressed. Rapamycin does affect how sensory information is processed to elicit sensory avoidance behaviors. The cardiac physiology appears to be slighted altered, potentially in how intracellular Ca2+ might be handled based on altered response to 5-HT. The implications for this work are in the realm of human health, though some studies have proposed rapamycin as a cancer treatment in other mammals (Enjoji et al., 2015). Clinical use of rapamycin and other mTOR inhibitors as a cancer and immunotherapy treatment is becoming popular, and there is mounting evidence of the mTOR pathway's importance in neurologic and psychiatric disease. Though it has shown promise as a treatment, the possible side effects and negative consequences have yet to be thoroughly explored.

## MATERIALS AND METHODS

This project used a three-pronged experimental approach: life cycle quantification, behavioral assays and electrophysiological measurements. To address the effect of mTOR inhibition on life cycle alteration, time to pupation was measured in *D. melanogaster* (Canton S strain) larvae with varying dosages of rapamycin treatment. The effect on muscle development was assessed in two ways. First, behavioral assays were performed on rapamycin-fed larvae to determine if treatment caused abnormal behavior, possibly due to improperly developed muscles. Secondly, electrophysiological measurements of the resting membrane potential, amplitude of EJPs and membrane input resistance were measured in larvae fed various dosages of rapamycin to quantify muscle development and health. To ascertain any effect on cardiac function by rapamycin treatment, rapamycin-fed larvae were dissected and heart rate quantified before and after modulation with 5-HT.

### Rapamycin feeding procedure

For each of the experimental approaches, the same rapamycin feeding procedure was used, the only difference being the duration of exposure. Half a gram of pre-mixed cornmeal food was placed in plastic vials. Control food was prepared with the addition of 150 µl of ethanol solvent, while the experimental food vials were supplemented with varying dosages of rapamycin dissolved in 150 µl of ethanol. A 48-h evaporation period followed food supplementation to allow for the ethanol to evaporate out of the food. Second instar larvae were then removed from normal cornmeal food media and divided into control and experimental groups before being added to the appropriate vials.

### Life cycle quantification

Food was prepared following the above procedure to produce the following: ethanol only for a control, 10 µM, 30 µM and 100 µM rapamycin. After the addition of 15 first instar larvae per vial (*n*=15 for all groups), vials were placed in a room with constant temperature (21°C) and normal day and night light cycles (12 h light 12 h dark). Vials were monitored daily for the formation of new pupae, which is characterized by the formation of a brown, immobile casing on the vial wall. Upon the formation of a new pupa, a mark was placed on the exterior of the vial to indicate which pupae had already been counted. The date and time of new pupae's first observation were also documented. Vials were assessed daily to determine whether water needed to be added to prevent the food from drying out and to ensure that the larvae were still alive in the food, in accordance with protocols developed in previous studies (Potter et al., 2016). Vials were monitored for 11 days after the formation of the first control pupa. After the final pupa documentation, contents of each vial were examined for living and dead larvae.

Time to pupation data were graphed as percentage pupated versus time. Each treatment (control and the three rapamycin treatments) consisted of 15 larvae, so percentages pupated were calculated using the total number of pupa out of 15 for each time point. Relative percent pupated over time was also determined for ease in comparing delays in development. The time of last data collection was used as a censored data point for larvae that were alive but had not yet pupated at the end of the pupation data collection period.

### Behavioral assays

Food was prepared following the above procedure to produce food vials containing the ethanol as a control, 200 µM, and 500 µM rapamycin treatments. After the addition of approximately 60 third instar larvae, vials were placed in a room with constant temperature and normal day and night light cycles. After a 24-h feeding, larvae were removed from the food to perform behavioral assays. Assays performed included mouth-hook movements (MHM) and body-wall movements (BWM). The MHM assay consisted of counting the number of times the larvae move their mouth-hooks in 1 min in a yeast paste solution. The BWM assay consisted of counting the number of times the larvae moved their body-wall in 1 min on an apple juice agar plate. All assays were performed under a light microscope, and count assays were conducted by eye.

The results of the mouth-hook and body-wall assays were rates (number of occurrences per minute) for each tested larvae in each group. For both assays, the control group consisted of 36 larvae (*n*=36), and the 200 µM rapamycin treatment had 38 larvae (*n*=38). The 500 µM rapamycin treatment had 58 larvae for the mouth-hook assay (*n*=58) and 59 larvae for the body-wall assay (*n*=59). The statistical analysis for both assays consisted of ANOVA to test the differences between the means of all three groups.

The response to touch (mechanical stimuli) of the larvae fed rapamycin was the same as described previously in detail (Titlow et al., 2014). In brief, individual larvae were placed on an 8 cm agar dish (1% agar, 33% apple juice to stimulate crawling). Crawling larvae (early third instar) were prodded three times with an insect pin (Fine Science Instruments, 0.2 mm diameter), once on the head, abdomen, and then tail. This is referred to as the HAT assay. An observer recorded all behavioral responses evoked by the stimulus, e.g. no response (NR), pause, etc. See Titlow et al., (2014) for details on force calibration, methods of mechanical stimulation and details on the neural circuits recruited for this sensory-motor behavior. The results for the HAT assay were relative percentage of each response type within treatment level at each of the three body positions. The control group consisted of 36 larvae (*n*=36), the 200 µM rapamycin treatment had 38 larvae (*n*=38), and the 500µM rapamycin treatment had 57 larvae (*n*=57).

### Electrophysiology measurements

Food was prepared following the above procedure to produce food vials containing the ethanol control, 200 µM and 500 µM rapamycin treatments. After the addition of approximately 15 larvae, vials were placed in a room with constant temperature (21°C) and normal day and night light cycles (12 h light 12 h dark). After an acute 24-h feeding, larvae were removed from the food to perform electrophysiology measurements. Each larva was dissected along the mid-dorsal longitudinal axis and pinned flat. A glass slide with magnetic tape affixed to one side was utilized as the dissecting dish. Each preparation could be viewed with transmitted light by cutting a circular hole in the magnetic tape, thus exposing the underlying glass. Bent dissecting pins were affixed to small metal bases. This arrangement allowed the pins to be easily maneuvered along the magnetic tape while also keeping the specimen fixed in saline slide (preparation of the recording dishes shown in video format in Cooper et al., 2009). The physiological saline used contained (in mM) 1.0 CaCl_2_.2H_2_O, 70 NaCl, 5 KCl, 10 NaHCO_3_, 5 trehalose, 115 sucrose, 25 *N,N*-bis(2- hydoxyethyl)-2-aminoethanesulfonic acid (BES) (de Castro et al., 2014). All chemicals were obtain from Sigma-Aldrich.

Recordings were collected at room temperature (20–21°C). Recordings were obtained with intracellular microelectrodes filled with 3 M KCl having a resistance of 30–60 mΩ. Responses were recorded with a 1X LU head stage and an Axoclamp 2A amplifier. The recording techniques for intracellular (EJPs) measures have been previously described (Dasari and Cooper, 2004a,b; Sparks et al., 2004; Stewart et al., 1994). The compound amplitude of EJP elicited by Is and Ib motor nerve terminals in segment 3 of muscle m6 was monitored (Kurdyak et al., 1994; Ruffner et al., 1999). The EJP amplitudes were obtained at 0.5-Hz simulation frequency. Facilitation/depression was measured by an index of the ratio of the peak amplitude of the fourth EJP within the train to the first EJP amplitude within the same stimulus train. A unitary value of 1 was subtracted from the ratio to provide a facilitation index (FI). This is to ensure that if no facilitation is present (i.e. the amplitudes of the responses are the same), FI will be zero. If FI is negative, then by definition this will produce short-term depression and if the value is above zero then, this will produce STF. Electrical signals were recorded to a laboratory computer via a PowerLab/4 s interface (ADInstruments, USA). All events were measured and calibrated with Scope software version 3.5.4 (ADInstruments, USA). Averages of 10–20 traces of evoked EPSPs were made to obtain an overall average as presented for the nerve terminals. The average for each preparation in each treatment group was used to calculate the mean and standard error.

In addition to resting membrane potential and EJP amplitudes, membrane resistance was also determined for another set of 200 µM rapamycin-fed and control larvae with nine larvae per group. This procedure consisted of injecting negative and positive square pulse current into muscle preparations and measuring changes in membrane potential. The dissections were performed in the same manner as described above, and the same saline and electrical equipment parameters were utilized. Currents utilized were −3 nA, −2.5 nA, −2 nA, −1 nA, 1 nA, 2 nA and 2.5 nA, and change in membrane potential was measured for each current injection during the plateau phase of the potential (∼250 ms). Plots of injected current versus potential change were made for each treatment condition and lines of fit applied to each of the nine preparations per group. Membrane resistance was then determined by applying Ohm's law to the fitted lines. Data for resting membrane potential was collected from five larvae in each of the three conditions, with multiple recordings per larva averaged into a single recording (*n*=5). EJP magnitude was also collected from five larvae in each of the three conditions, with multiple recordings per larva averaged into a single recording (*n*=5). Both variables were statistically analyzed using ANOVA comparing means between the three groups.

Initial data from the second electrophysiological protocol consisted of injected currents and recording the corresponding potential change, as described above. Larvae in both the control and 200 µM rapamycin groups were examined (*n*=9 each condition). The actual value of interest is membrane resistance, which was calculated using the lines of fit to the collected data and an application of Ohm's law. A two sample *t*-test was then applied to the membrane resistances in both groups to determine whether the means differed.

### Measures of heart rate

Food was prepared following the above procedure and approximately 15 larvae were placed in vials containing prepared food. After an acute 24-h feeding, larvae were removed from the food to perform heart rate measurements. The general larval dissection technique to expose the larval heart tubes has been previously reported (Cooper et al., 2009). In brief, the larvae were dissected ventrally and pinned on four corners to expose the heart tube. The visceral organs were removed, keeping the heart tube intact. This dissection technique was previously used to directly assess pharmacological agents on the heart of *Drosophila* larvae (Desai-Shah et al., 2010; Majeed et al., 2014; Malloy et al., 2016). The dissection time was roughly 3–6 min, and each preparation was allowed to relax while bathed in saline for 3–5 min after dissection. A modified HL3 saline was used to maintain the *in situ* hearts and body-wall muscles [NaCl 70 mM, KCl 5 mM, MgCl2.6H2O 20 mM, NaHCO3 10 mM, Trehalose 5 mM, sucrose 115 mM, BES 25 mM, and CaCl2.2H2O 1 mM; pH 7.1 (de Castro et al., 2014)]. Salts for the saline and 5-HT were obtained from Sigma Chemical Company. The heart rates were measured while the heart was exposed to saline and then again 1 min after bathing media was exchanged with one containing 5-HT (100 nM). This concentration provides a significant response in increasing heart rate in the larvae (de Castro et al., 2019; Majeed et al., 2014; Zhu et al., 2016a,b).
